# Supplementary material for: A Low-power wearable acoustic device for accurate invasive arterial pressure monitoring
Source: Commun Med (Lond). 2023 May 20;3:70. doi: 10.1038/s43856-023-00296-8 (PMC10199919; doi:10.1038/s43856-023-00296-8)
Supplement: Supplementary file 2 — Description of Additional Supplementary Files [file 43856_2023_296_MOESM2_ESM.pdf]

Description of Additional Supplementary File

File Name: Supplementary Data 1

Description: This contains all the data used to generate the figures. Each tab in the excel spreadsheet contains the data for a different figure.
