# Supplementary material for: A Low-power wearable acoustic device for accurate invasive arterial pressure monitoring
Source: Commun Med (Lond). 2023 May 20;3:70. doi: 10.1038/s43856-023-00296-8 (PMC10199919; doi:10.1038/s43856-023-00296-8)
Supplement: Supplementary file 3 — Supplementary Information [file 43856_2023_296_MOESM3_ESM.pdf]

A low-power wearable acoustic device
for accurate invasive arterial pressure monitoring

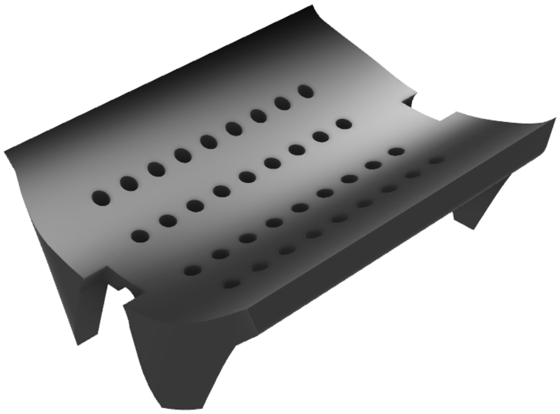

Supplementary Figure 1: **CAD Model for mounting the localization hardware onto a standard IV pole.** Hole cut outs are used to both mount our localization hardware and also to tie down the entire system to the IV pole.
